# Association of admission serum calcium level with left ventricular dysfunction in patients with acute coronary syndrome

**DOI:** 10.3389/fcvm.2022.1018048

**Published:** 2022-11-29

**Authors:** Hong Wang, Rongrong Wang, Junping Tian

**Affiliations:** ^1^Department of Endocrinology, Aerospace Center Hospital, Beijing, China; ^2^Department of Rheumatology, Aerospace Center Hospital, Beijing, China; ^3^Department of Cardiovascular Medicine, Beijing Tiantan Hospital, Capital Medical University, Beijing, China

**Keywords:** acute coronary syndrome, serum calcium, left ventricular dysfunction, left ventricular ejection fraction, B-type natriuretic peptide

## Abstract

**Background:**

The relationship between serum calcium and left ventricular function in patients with acute coronary syndrome (ACS) has not been explored. Our aim was to investigate the correlation of admission serum calcium with left ventricular dysfunction in ACS patients.

**Methods:**

In this cross-sectional study, 658 ACS patients who were admitted in the Department of Cardiovascular Disease from June 1st, 2019 to December 31st, 2019 were enrolled in the present study. Serum calcium and B-type natriuretic peptide (BNP) were measured at admission. Left ventricular ejection fraction (LVEF) was assessed using echocardiography. The correlation between admission serum calcium and left ventricular dysfunction was analyzed.

**Results:**

When stratified by serum calcium quartiles calculated from all patients, patients with lower serum calcium quartile showed a markedly higher BNP and lower LVEF (*P* < 0.05). Patients with LVEF ≤ 50% showed a significantly lower serum calcium and higher BNP compared to those with LVEF> 50% (*P* < 0.05). Admission serum calcium was positively correlated with LVEF (*P* < 0.01) but negatively correlated with BNP (*P* < 0.01). Multivariate logistic regression analysis showed that lower serum calcium (adjusted OR: 0.720, 95% CI: 0.519–0.997, *P* = 0.048) was independently associated with BNP **≥** 300 pg/ml in ACS patients. Using LVEF as a dependent variable, no significant correlation between low serum calcium and left ventricular systolic dysfunction was found in ACS patients.

**Conclusions:**

In patients with ACS, admission serum calcium was positively correlated with LVEF and negatively with BNP. Lower admission serum calcium was an independent risk factor for elevated BNP.

## Introduction

Cardiovascular disease is the major cause of death globally (1, 2). Acute coronary syndrome (ACS) represents a severe type of atherosclerotic cardiovascular disease. Studies reported that about 30–50% of ACS patients had left ventricular systolic dysfunction (3–5) which was an important determinant of poor outcome (3, 6, 7).

Serum calcium is a common monitored clinical biomarker. Calcium plays a critical role in excitation, contraction and relaxation of the myocardium (8, 9). Both clinical electrophysiology studies and preclinical experiments demonstrated that abnormal intracellular calcium concentration was a key biomarker of heart failure with preserved ejection fraction (10–12). Previous studies indicated that high level of serum calcium concentration was independently associated with heart failure (10–12). However, Wang et al. (13) found that low serum calcium was associated with left ventricular systolic dysfunction in patients with coronary artery disease (CAD).

The relationship between serum calcium and left ventricular function in ACS patients has not been investigated. Therefore, our aim was to explore the correlation between admission serum calcium and left ventricular dysfunction in ACS patients.

## Methods

### Study participants

Patients with ACS who were admitted to the Department of Cardiovascular Disease, Beijing Tiantan Hospital, Capital Medical University from June 1st, 2019 to December 31st, 2019 were screened. The ACS included unstable angina pectoris, acute ST-segment elevation myocardial infarction (STEMI) and non-ST-segment elevation myocardial infarction (non-STEMI) (14). The inclusion criteria were as follows: (1) age > 18 years; (2) ACS was confirmed by coronary angiography; (3) complete information of echocardiographic examination; (4) signed informed consent form. Patients with any of the following conditions were excluded: preexisting cardiomyopathy, endocarditis, severe valvular heart disease, cerebrovascular disease, liver dysfunction, chronic renal insufficiency, acute infection, known malignancies, thyroid diseases, parathyroid disorders, mal-absorption, bone disease, systemic immune disease, hemorrhagic diathesis or coagulation disorders. In total, 658 ACS patients were eligible for the study. The study was performed according to the Declaration of Helsinki and approved by the ethical committee of Beijing Tiantan hospital. Written informed consents were obtained from all patients or their legal representatives.

### Demographic and clinical data

Clinical data including height, weight, body mass index, blood pressure, heart rate, and Global Registry of Acute Coronary Events (GRACE) risk score were collected at admission. Medical history, history of smoking and drinking were also collected upon admission. GRACE score was calculated based on the following eight indexes: age, heart rate, systolic blood pressure, blood creatinine concentration, Killip classification, prehospital cardiac arrest, ST-segment deviation on electrocardiography, and cardiac enzyme elevation.

### Biochemical measurements

Fasting blood samples were obtained from the peripheral veins on the first morning after admission. Levels of serum calcium, phosphate, albumin, serum lipid, serum glucose and creatinine were measured by the Hitachi LABOSPECT 008 automatic biochemical analyzer (Hitachi Corporation, Japan). Glycosylated hemoglobin A1c (HbA1c), C-reactive protein (CRP), and B-type natriuretic peptide (BNP) were determined using routine methods. Abnormal elevated BNP was defined as ≥300 pg/ml (15–17). The troponin I (TnI), and creatine kinase isoenzymes MB (CK-MB) levels were also measured using the Architect system (Abbott Diagnostics).

### Coronary angiography

Coronary angiography was carried out *via* the radial or femoral artery approach according to the standard Judkins technique in ACS patients. CAD was defined as having a single or multiple coronary artery stenosis with at least 50% diameter reduction according to the American College of Cardiology/American Heart Association lesion classification (18). Based on the results of coronary angiography, CAD severity was estimated by the number of coronary artery lesions and classified as three groups: single-vessel disease, double-vessel disease, triple-vessel disease. And left main lesion was equivalent to triple-vessel disease (19).

### Left ventricular systolic function assessment

Echocardiography was performed with a Vivid 7 Dimension equipped with a multifrequency transducer (GE Healthcare, Vingmed, Norway) within 24 h after admission. Left ventricular systolic function was evaluated by left ventricular ejection fraction (LVEF). LVEF was measured using the Simpson's method based on two-dimensional echocardiography in all patients. Low LVEF was defined as LVEF ≤ 50% (20, 21).

### Statistical analysis

Normality of the distribution of the studied variables was assessed by normal probability plots and one-sample Kolmogorov–Smirnov test. Continuous variables were presented as mean ± standard deviation (SD) or median (interquartile range), as appropriate. Differences in normally distributed variables were determined by independent-samples *t*-test or one-way ANOVA, while the non-parametric Mann-Whitney *U*-test or Kruskal-Wallis test was used for the variables not normally distributed. Categorical variables were given as ratio or percentage. The Chi-square test was applied for categorical variables. Pearson correlation was performed to determine the association between serum calcium and left ventricular function. Multivariate logistic regression analyses were used to examine the relationship between left ventricular dysfunction based on BNP ≥ 300 pg/ml or LVEF ≤ 50% and related factors, after adjustment for potential confounders, including age, gender, biochemical parameters and GRACE score. The odds ratio (OR) and 95% confidence interval (95% CI) were reported separately. A two-tailed *P*-value < 0.05 was considered as statistically significant difference.

The data were analyzed with SPSS version 26 (SPSS for Windows, IBM Corp., USA).

## Results

### Comparisons of clinical characteristics of ACS patients according to serum calcium quartiles

In total, 658 ACS patients were included, with a mean age of 62.0 ± 10.1 years, (range: 23–85 years). Four hundred and seventy-seven participants (72.5%) were males. The average serum calcium level was 2.27 ± 0.10 mmol/l. The average LVEF was 60.7 ± 6.8% and the proportion of LVEF ≤ 50 was 9.3%.

All patients were divided into four groups according to serum calcium quartiles: Quartile 1 (serum calcium level ≤ 2.19 mmol/L), Quartile 2 (2.20–2.25 mmol/L), Quartile 3 (2.26–2.32 mmol/L), and Quartile 4 (≥2.33 mmol/L). The biochemical indexes and clinical data of ACS patients in the different groups were shown in Table 1. When stratified by serum calcium quartiles, the mean age, proportion of acute myocardial infarction, troponin I at admission, BNP and GRACE score were significantly higher in Quartile 1 group as compared with Quartile 3 group and Quartile 4 group (*P* < 0.01). At the same time, the patients in Quartile 2 group were older than those in Quartile 4 group and also had higher troponin I at admission, BNP and GRACE score than those in Quartile 3 group and Quartile 4 group (*P* < 0.05). However, patients in Quartile 1 group showed an obviously lower LVEF than those in Quartile 3 group and Quartile 4 group (*P* < 0.01). A lower triglycerides and total cholesterol were found in Quartile 1 group than those in Quartile 4 group (*P* < 0.01). And body mass index was also lower in Quartile 1 group than that in Quartile 2 group and Quartile 3 group (*P* < 0.01). With increasing serum calcium, serum albumin and serum phosphorus showed a significant increase (*P* < 0.05). No differences in gender, previous history, blood pressure, creatinine or number of coronary artery lesions were observed among four groups.

**Table 1:** Comparisons of clinical characteristics of ACS patients according to serum calcium quartiles.

**Variables**	**Total**	**Quartile 1 (≤ 2.19)**	**Quartile 2 (2.20–2.25)**	**Quartile 3 (2.26–2.32)**	**Quartile 4 (≥2.33)**	***P*-value**
Number	658	146	154	193	165	–
Age (years)	62.0 ± 10.1	64.7 ± 9.0	63.1 ± 9.5	61.1 ± 9.8^*^	59.7 ± 11.3^*^^#^	**< 0.001**
Gender (male/female)	477/181	114/32	104/50	146/47	113/52	0.090
BMI (kg/m^2^)	25.55 ± 2.81	24.96 ± 2.58	25.81 ± 2.86^*^	25.86 ± 2.97^*^	25.47 ± 2.68	**0.016**
Previous history
Coronary artery disease (%)	275 (41.8)	60 (41.1)	61 (39.6)	83 (43.0)	71 (43.0)	0.908
Hypertension (%)	456 (69.3)	93 (63.7)	108 (70.1)	135 (69.9)	120 (72.7)	0.369
Diabetes (%)	256 (38.9)	53 (36.3)	61 (39.6)	74 (38.3)	68 (41.2)	0.839
Stroke (%)	73 (11.1)	15 (10.3)	21 (13.6)	19 (9.8)	18 (10.9)	0.701
Smoking (%)	378 (57.4)	85 (58.2)	84 (54.5)	115 (59.6)	94 (57.0)	0.815
Drinking (%)	310 (47.1)	68 (46.6)	66 (42.9)	97 (50.3)	79 (47.9)	0.585
Diagnosis						**0.003**
AMI (%)	142 (21.6)	47 (32.2)	34 (22.1)	34 (17.6)^*^	27 (16.4)^*^	
Unstable angina pectoris (%)	516 (78.4)	99 (67.8)	120 (77.9)	159 (82.4)	138 (83.6)	
Systolic BP (mmHg)	128.9 ± 17.1	126.8 ± 19.2	129.7 ± 16.6	129.7 ± 16.7	128.9 ± 15.9	0.389
Diastolic BP (mmHg)	77.2 ± 10.6	77.1 ± 10.9	77.0 ± 10.5	76.9 ± 10.4	77.7 ± 10.8	0.908
Heart rate (beats per min)	71.7 ± 9.3	71.8 ± 9.9	71.0 ± 8.6	71.6 ± 9.2	72.4 ± 9.5	0.594
Troponin I at admission (ng/mL)	0.005 (0.002, 0.066)	0.011 (0.003, 0.876)	0.008 (0.002, 0.087)	0.004 (0.002, 0.018)^*^^∧^	0.004 (0.001, 0.018)^*^^#^	**< 0.001**
Hemoglobin (g/L)	140.9 ± 15.5	138.1 ± 16.9	141.3 ± 15.5	141.1 ± 14.5	142.5 ± 15.3	0.083
Serum albumin (g/L)	40.3 ± 3.0	38.1 ± 2.8^*^	39.6 ± 2.3^#^	40.5 ± 2.3^&^	42.5 ± 2.7	**< 0.001**
Serum calcium (mmol/L)	2.27 ± 0.10	2.13 ± 0.05^*^	2.23 ± 0.02^#^	2.29 ± 0.02^&^	2.39 ± 0.06	**< 0.001**
Serum phosphorus (mmol/L)	1.13 ± 0.19	1.05 ± 0.20^*^	1.11 ± 0.17	1.16 ± 0.18^∧^	1.18 ± 0.17^#^	**< 0.001**
Serum glucose (mmol/L)	4.91 (4.34, 6.04)	4.75 (4.26, 5.76)	4.89 (4.37, 6.16)	4.98 (4.36, 5.96)	5.08 (4.40, 6.53)	0.123
Triglycerides (mmol/L)	1.46 (1.04, 2.01)	1.33 (0.95, 1.86)	1.41 (1.01, 1.92)	1.48 (1.04, 1.97)	1.64 (1.22, 2.24)^*^^#^	**0.001**
Total cholesterol (mmol/L)	3.89 ± 0.96	3.75 ± 0.84	3.80 ± 0.93	3.89 ± 0.96	4.08 ± 1.07^*^^#^	**0.013**
LDL-C (mmol/L)	2.43 ± 0.89	2.34 ± 0.76	2.39 ± 0.86	2.41 ± 0.91	2.56 ± 1.00	0.157
HDL-C (mmol/L)	1.01 ± 0.25	1.01 ± 0.28	0.97 ± 0.20	1.03 ± 0.27	1.03 ± 0.25	0.125
Serum uric acid (μmol/L)	349.6 ± 86.0	333.4 ± 78.8	350.0 ± 88.3	353.4 ± 77.5	359.3 ± 97.5	0.053
Creatinine (μmol/L)	68.1 ± 17.1	69.6 ± 20.5	67.5 ± 15.7	68.5 ± 16.6	66.9 ± 15.5	0.527
Glycosylated hemoglobin A1C (%)	6.72 ± 1.38	6.50 ± 1.15	6.70 ± 1.32	6.84 ± 1.46	6.79 ± 1.50	0.136
BNP (pg/mL)	45.0 (22.9, 115.7)	69.3 (32.0, 193.2)	48.5 (25.8, 136.6)	39.4 (18.6, 90.5)^*^^∧^	36.7 (16.4, 80.2)^*^^#^	**< 0.001**
LVEF (%)	60.7 ± 6.8	59.2 ± 7.0	60.5 ± 7.4	61.5 ± 6.6^*^	61.3 ± 5.9^*^	**0.012**
GRACE score	113.6 ± 23.6	121.0 ± 25.2	116.6 ± 21.0^#^	109.6 ± 23.3^*^	108.9 ± 22.9^*^	**< 0.001**
Number of coronary artery lesions	2 (1, 3)	2 (1, 2)	2 (1, 3)	2 (1, 3)	2 (1, 3)	0.562

BMI, body mass index; AMI, acute myocardial infarction; BP, blood pressure; LDL-C, low density lipoprotein cholesterol; HDL-C, high density lipoprotein cholesterol; BNP, B-type natriuretic peptide; LVEF, left ventricular ejection fraction; GRACE, Global Registry of Acute Coronary Events.

* Q1 vs. Q2, Q1 vs. Q3, Q1 vs. Q4, *P* < 0.01.

# Q2 vs. Q3, Q2 vs. Q4, *P* < 0.01.

∧ Q2 vs. Q3, *P* < 0.05.

& Q3 vs. Q4, *P* < 0.01.

The bold values indicate statistical differences.

### Comparisons of clinical characteristics of ACS patients according to LVEF

The ACS patients were categorized into two groups according to LVEF, with one group of ACS patients with lower LVEF (LVEF ≤ 50%, *n* = 61) and one group with higher LVEF (LVEF > 50%, *n* = 597). Seen from Table 2, the male, smoking, proportion of acute myocardial infarction, heart rate, troponin I at admission, creatinine, BNP, and GRACE score were obviously higher in lower LVEF group than in higher LVEF group (*P* < 0.05). As compared to those with higher LVEF, the patients with lower LVEF had a significantly decreased serum albumin, serum calcium and phosphorus (*P* < 0.05). The age, blood pressure, serum lipid, or number of coronary artery lesions didn't differ between both groups.

**Table 2:** Comparisons of clinical characteristics of ACS patients according to LVEF.

**Variables**	**Lower LVEF group (≤ 50%)**	**Higher LVEF group (>50%)**	***P*-value**
No.	61	597	
Age (years)	61.3 ± 11.5	62.1 ± 10.0	0.569
Gender (male/female)	53/8	424/173	**0.008**
BMI (kg/m^2^)	25.42 ± 2.96	25.56 ± 2.79	0.709
Previous history
Coronary artery disease (%)	31 (50.8)	244 (40.9)	0.133
Hypertension (%)	38 (62.3)	418 (70.0)	0.213
Diabetes (%)	27 (44.3)	229 (38.4)	0.368
Stroke (%)	10 (16.4)	63 (10.6)	0.167
Smoking (%)	47 (77.0)	331 (55.4)	**0.001**
Drinking (%)	36 (59.0)	274 (45.9)	0.051
Diagnosis			**< 0.001**
AMI (%)	35 (57.4)	107 (17.9)	
Unstable angina pectoris (%)	26 (42.6)	490 (82.1)	
Systolic BP (mmHg)	126.2 ± 21.2	129.1 ± 16.6	0.301
Diastolic BP (mmHg)	77.5 ± 13.3	77.1 ± 10.3	0.817
Heart rate (beats per min)	75.7 ± 14.3	71.3 ± 8.5	**0.022**
Troponin I at admission (ng/mL)	0.076 (0.012, 16.914)	0.004 (0.002, 0.040)	**< 0.001**
Hemoglobin (g/L)	141.7 ± 19.1	140.8 ± 15.1	0.728
Serum albumin (g/L)	39.4 ± 3.2	40.3 ± 2.9	**0.021**
Serum calcium (mmol/L)	2.24 ± 0.10	2.27 ± 0.10	**0.043**
Serum phosphorus (mmol/L)	1.08 ± 0.21	1.13 ± 0.19	**0.028**
Serum glucose (mmol/L)	5.30 (4.32, 6.74)	4.89 (4.35, 5.98)	0.181
Triglycerides (mmol/L)	1.30 (1.01, 1.95)	1.46 (1.05, 2.02)	0.413
Total cholesterol (mmol/L)	4.00 ± 1.05	3.88 ± 0.96	0.347
LDL-C (mmol/L)	2.55 ± 0.95	2.42 ± 0.89	0.272
HDL-C (mmol/L)	1.02 ± 0.27	1.01 ± 0.25	0.900
Serum uric acid (μmol/L)	365.2 ± 85.6	348.0 ± 86.0	0.137
Creatinine (μmol/L)	72.2 ± 15.9	67.7 ± 17.1	**0.049**
Glycosylated hemoglobin A1C (%)	6.98 ± 1.58	6.69 ± 1.36	0.123
BNP (pg/mL)	147.8 (37.6, 354.4)	42.8(21.6, 100.4)	**< 0.001**
GRACE score	128.9 ± 29.5	112.0 ± 22.4	**< 0.001**
LVEF (%)	44.3 ± 6.1	62.4 ± 4.1	**< 0.001**
Number of coronary artery lesions	2 (1, 3)	2 (1, 3)	0.185

BMI, body mass index; AMI, acute myocardial infarction; BP, blood pressure; LDL-C, low density lipoprotein cholesterol; HDL-C, high density lipoprotein cholesterol; BNP, B-type natriuretic peptide; LVEF, left ventricular ejection fraction; GRACE, Global Registry of Acute Coronary Events.

The bold values indicate statistical differences.

### Correlations of serum calcium with left ventricular function in ACS patients

In Table 3, Pearson correlation analysis found that admission serum calcium level showed a significantly positive correlation with hemoglobin, serum albumin, serum glucose, serum uric acid, serum phosphorus, triglycerides, total cholesterol, low density lipoprotein cholesterol, and LVEF (*P* < 0.05). And serum calcium was significantly and negatively associated with age, BNP, troponin I at admission, and GRACE score (*P* < 0.001).

**Table 3:** Correlations of serum calcium in ACS patients.

**Variables**	***r*-value**	***P*-value**	**Variables**	***r*-value**	***P*-value**
Age (years)	−0.186	**< 0.001**	Creatinine (μmol/L)	−0.053	0.176
BMI (kg/m^2^)	0.065	0.097	Triglycerides (mmol/L)	0.149	**< 0.001**
Systolic BP (mmHg)	0.063	0.106	Total cholesterol (mmol/L)	0.131	**0.001**
Diastolic BP (mmHg)	0.036	0.358	LDL-C (mmol/L)	0.092	**0.019**
Hemoglobin (g/L)	0.101	**0.010**	HDL-C (mmol/L)	0.067	0.084
Serum albumin (g/L)	0.592	**< 0.001**	BNP (pg/mL)	−0.155	**< 0.001**
Serum glucose (mmol/L)	0.089	**0.023**	Troponin I at admission (ng/mL)	−0.171	**< 0.001**
Serum uric acid (μmol/L)	0.104	**0.008**	LVEF (%)	0.115	**0.003**
Serum phosphorus (mmol/L)	0.264	**< 0.001**	GRACE score	−0.230	**< 0.001**

BMI, body mass index; BP, blood pressure; LDL-C, low density lipoprotein cholesterol; HDL-C, high density lipoprotein cholesterol; BNP, B-type natriuretic peptide; LVEF, left ventricular ejection fraction; GRACE, Global Registry of Acute Coronary Events.

The bold values indicate statistical differences.

### Multivariate logistic regression in ACS patients

In multivariate logistic regression analysis, BNP was defined as 1 when BNP ≥ 300 pg/ml, and BNP was defined as 0 when BNP < 300 pg/ml. LVEF was considered as 1 when LVEF ≤ 50%, and LVEF was considered as 0 when LVEF > 50%. Male was defined as 1 while female was as 0. Multivariate backward stepwise logistic regression analysis was used to explore the independent factors associated with BNP ≥ 300 pg/ml or LVEF ≤ 50%.

In model 1, age, gender and serum calcium quartile were used as the independent variables. The data in Table 4 exhibited that age (OR: 1.049, 95% CI: 1.016–1.083, *P* = 0.003) and lower serum calcium quartile (OR: 0.569, 95% CI: 0.429–0.754, *P* < 0.001) were independently associated with BNP ≥ 300 pg/ml after adjustment for potential confounding factors in ACS patients. In contrast, male (OR: 2.640, 95% CI: 1.228–5.678, *P* = 0.013) and lower serum calcium quartile (OR: 0.796, 95% CI: 0.623–1.016, *P* = 0.066) correlated with LVEF ≤ 50%.

**Table 4:** Backward stepwise multivariate logistic regression in ACS patients.

**Variables**	**BNP**				**LVEF**			
	**β**	**OR**	**95% CI**	***P*-value**	**β**	**OR**	**95% CI**	***P*-value**
**Model 1**
Age (years)	0.048	1.049	1.016–1.083	**0.003**				0.824
Gender				0.213	0.971	2.640	1.228–5.678	**0.013**
Serum calcium quartile	−0.564	0.569	0.429–0.754	**< 0.001**	−0.228	0.796	0.623–1.016	0.066
**Model 2**
Age (years)				0.388	−0.071	0.931	0.900–0.965	**< 0.001**
Gender				0.956	0.904	2.469	1.095–5.565	**0.029**
Serum calcium quartile	−0.329	0.720	0.519–0.997	**0.048**				0.402
Serum albumin (g/L)	−0.104	0.901	0.802–1.012	0.080				0.578
Creatinine (μmol/L)	0.020	1.020	1.005–1.035	**0.010**				0.943
Serum phosphorus (mmol/L)				0.120				0.780
GRACE score	0.041	1.042	1.027–1.057	**< 0.001**	0.049	1.050	1.035–1.066	**< 0.001**

In Model 1, age, gender and serum calcium quartile were used as the independent variables.

In Model 2, age, gender, serum calcium quartile, serum phosphorus, serum albumin, creatinine and GRACE score were included as the independent variables.

The bold values indicate statistical differences.

In model 2, serum phosphorus, serum albumin, creatinine and GRACE score were also included in addition to those used in model 1. Multivariate backward stepwise logistic regression showed that lower serum calcium quartile (OR: 0.720, 95% CI: 0.519–0.997, *P* = 0.048), lower serum albumin (OR: 0.901, 95% CI: 0.802–1.012, *P* = 0.080), increased creatinine (OR: 1.020, 95% CI: 1.005–1.035, *P* = 0.010) and GRACE score (OR: 1.042, 95% CI: 1.027–1.057, *P* < 0.001) were independently correlated with BNP ≥ 300 pg/ml after adjusting for other potential confounders in ACS patients. When LVEF was used as the dependent variable, male (OR: 2.469, 95% CI: 1.095–5.565, *P* = 0.029), age (OR: 0.931, 95% CI: 0.900–0.965, *P* < 0.001) and increased GRACE score (OR: 1.050, 95% CI: 1.035–1.056, *P* < 0.001) were independently associated with LVEF ≤ 50%.

## Discussion

The present study analyzed the correlation between the level of admission serum calcium and left ventricular dysfunction in ACS patients. Admission serum calcium was positively correlated with LVEF and negatively correlated with BNP. Low level of serum calcium was independently associated with BNP ≥ 300 pg/ml after adjusting for other potential confounders. No significant association between low level of serum calcium and left ventricular systolic dysfunction was detected.

Previous studies investigated the correlation between serum calcium and left ventricular function and found that patients with low concentration of serum calcium were more likely to have LVEF < 50% (22). And patient with established heart failure presented low level of serum calcium concentration (13, 23). Wang et al. (13) suggested that low serum calcium was independently associated with left ventricular systolic dysfunction in CAD patients with and without acute myocardial infarction. Batra and Agarwal (24) observed severe hypocalcemia and increased BNP in a patient with hypocalcemic cardiomyopathy and severe heart failure. Our results were consistent with these previous findings.

Other studies, on the contrary, showed different results. Li et al. (11) indicated that an increase in serum calcium concentration was correlated with an increased risk of heart failure with preserved ejection fraction in patients with type 2 diabetes mellitus. Additionally, Lutsey et al. (10) also demonstrated that baseline high serum calcium was independently correlated with greater risk of incident heart failure in this population-based cohort.

Many reasons may contribute to these different observations. It is well-known that serum calcium is affected by many factors, such as diseases, drugs and measurement techniques. In aforementioned studies, patients with diabetes mellitus or communities population from the Atherosclerosis Risk in Communities (ARIC) cohort were recruited. But severely ill patients or patients with history of LVEF < 50% were excluded. In contrast, ACS patients who were severely ill or in emergency condition were enrolled in our study. Additionally, albumin-adjusted serum calcium concentration was used in the previous study while admission serum calcium was measured in this study. By multivariate logistic regression analysis, low serum calcium was independently correlated with elevated BNP after adjustment for potential confounding factors such as age, serum albumin, and so on in ACS patients.

BNP and LVEF are objective indexes for assessment of left ventricular function (17, 25). To the best of our knowledge, this is the first study to evaluate the association between the level of admission serum calcium and left ventricular function in ACS patients. We demonstrated that low serum calcium quartile was independently associated with elevated BNP and serum calcium level was positively correlated with LVEF. However, no significant association of serum calcium with left ventricular systolic dysfunction was observed. The potential reason includes the small number of patients with LVEF ≤ 50%. Meanwhile, we noticed that serum calcium level was no longer correlated with the LVEF ≤ 50% when adjusted by GRACE score. Killip classification is used to stratify patients according to the severity of their post-myocardial infarction heart failure and included in the calculation of GRACE score, which may cause the futile correlation. By contrast, GRACE score was not included in the regression model in Wang et al.'s study (13).

The possible mechanism of the correlation between serum calcium and left ventricular dysfunction is unclear. Serum calcium ions play a vital role in many metabolic and regulatory processes associated with cardiovascular disease, such as platelet adhesion and aggregation, blood coagulation, enzymatic activity, myocardial excitation, contraction and relaxation (8, 9). In view of the roles of serum calcium in myocardial excitation-contraction coupling and cardiac electrophysiologic effect, abnormalities of serum calcium may cause disarrangement of calcium homeostasis in the cytoplasm of cardiomyocytes and lead to myocardial dysfunction (22). On the other side, hypocalcemia reduces renal sodium excretion (26), thus contributes to fluid overload and decreased myocardial contractility, as demonstrated by decreased left ventricular work index (9, 27). In addition, decreased serum calcium was correlated with various common cardiovascular risk profiles, such as hypertension, dyslipidemia, and so on (8).

This study had some limitations. First, it was a single-center, observational study, whether similar results are obtained in other patient categories remains to be investigated. Second, the current study included a relatively small number of patients with LVEF ≤ 50%. Third, recent studies (28, 29) found that high curvatures and branching of coronary arteries were associated with the risk of development of atherosclerosis and plaque formation, however, coronary curvatures and branching were not considered in our study. ACS patients with non-obstructive coronary artery were not included. Fourth, serum calcium measurement was performed on a single occasion after admission. Serum calcium levels might vary over time, therefore a longitudinal study is required. Finally, the pathophysiologic mechanism of the association between hypocalcaemia and left ventricular dysfunction was not investigated in the present study. However, with the explorative results generated from this study, a further study will be conducted.

In summary, admission serum calcium was positively correlated with LVEF and negatively with BNP in ACS patients. Low admission serum calcium was an independent biomarker for elevated BNP.

## Data availability statement

The raw data supporting the conclusions of this article will be made available by the authors, without undue reservation.

## Ethics statement

The studies involving human participants were reviewed and approved by the Ethical Committee of Beijing Tiantan Hospital. The patients/participants provided their written informed consent to participate in this study.

## Author contributions

HW contributed to assembly of data, performed data analysis and interpretation, and wrote the manuscript. RW carried out the studies, collected and analyzed data, and participated in manuscript writing. JT conceived and designed this study, provided administrative support, collected and analyzed data, and wrote the manuscript. All authors have read and approved the final manuscript.

## Funding

This work was supported by the Basic and Clinical Research Collaboration Programme (15JL55), Capital Medical University.

## Conflict of interest

The authors declare that the research was conducted in the absence of any commercial or financial relationships that could be construed as a potential conflict of interest.

## Publisher's note

All claims expressed in this article are solely those of the authors and do not necessarily represent those of their affiliated organizations, or those of the publisher, the editors and the reviewers. Any product that may be evaluated in this article, or claim that may be made by its manufacturer, is not guaranteed or endorsed by the publisher.
